# Contrasting Effects of Adipokines on the Cytokine Production by Primary Human Bronchial Epithelial Cells: Inhibitory Effects of Adiponectin

**DOI:** 10.3389/fphar.2020.00056

**Published:** 2020-02-18

**Authors:** Hélène Salvator, Stanislas Grassin-Delyle, Emmanuel Naline, Marion Brollo, Caroline Fournier, Louis-Jean Couderc, Philippe Devillier

**Affiliations:** ^1^Laboratory of Research in Respiratory Pharmacology–UPRES EA220, UFR Sciences de la Santé Simone Veil, Université Paris-Saclay, Suresnes, France; ^2^Department of Respiratory Diseases, Hôpital Foch, Suresnes, France; ^3^Mass Spectrometry Platform & INSERM UMR1173, UFR Sciences de la Santé Simone Veil, Université Versailles Saint Quentin en Yvelines, Université Paris-Saclay, Montigny-le-Bretonneux, France

**Keywords:** adiponectin, obesity, human bronchial epithelial cell, receptor, cytokine

## Abstract

**Background:**

Obesity is associated with an elevated risk of respiratory infections and inflammatory lung diseases. The objective was to investigate (i) the effects of adipokines (adiponectin (APN), leptin, chemerin, and visfatin) on the production of cytokines by unstimulated and poly(I:C)- and TNF-α-activated human primary bronchial epithelial cells (hBECs), (ii) the cells’ expression of the APN receptors (AdipoR1 and AdipoR2), and (iii) the cells' production of APN.

**Methods:**

The hBECs were isolated from patients undergoing surgery for lung carcinoma. The cells were then cultured with human recombinant adipokines in the absence or presence of TNF-α or poly(I:C) for 24 h. Supernatant levels of cytokines (IL-6, CCL2, CCL5, CCL20, CXCL1, CXCL8) and APN were measured using ELISAs. The mRNA levels of AdipoR1 and AdipoR2 in hBECs were determined using a real-time quantitative PCR.

**Results:**

Of the four adipokines tested, only APN significantly influenced the basal production and the TNF-α poly(I:C)-induced production of cytokines by hBECs. APN (3-30 µg.ml^-1^) was associated with greater basal production of IL-6, CCL20, and CXCL8, lower basal production of CCL2 and CXCL1 and no difference in CCL5 production. APN inhibited the poly(I:C)-induced production of these five cytokines and the TNF-α-induced production of CCL2 and CXCL1. AdipoR1 and AdipoR2 were both expressed in hBECs. In contrast to human bronchial explants, isolated hBECs did not produce APN.

**Conclusions:**

The APN concentrations are abnormally low in obese individuals, and this fall may contribute to the susceptibility to viral lung infections and the severity of these infections in obese individuals.

## Introduction

Worldwide, about 800 million individuals are obese, and more than 1.5 billion are overweight. A high body mass index (BMI) is a risk factor for an expanding set of chronic diseases (WHO, 2017; NCD Risk Factor Collaboration (NCD-RisC), 2017; GBD 2015 Obesity Collaborators et al., 2017). Furthermore, many obese patients suffer from respiratory symptoms. In particular, obesity contributes to a high respiratory tract infection burden (Dixon and Peters, 2018; Maccioni et al., 2018), and was notably identified as a risk factor for severe influenza during the 2009 H1N1 pandemic (Morgan et al., 2010; Salvator et al., 2011; Van Kerkhove et al., 2011). Furthermore, epidemiological studies have demonstrated that chronic bronchial diseases (asthma and COPD) are more likely to occur in obese individuals and that obese people experience more severe symptoms, have worse quality of life, and use more healthcare resources (Boulet et al., 2012). A causal relationship is suggested by the fact that obesity usually precedes asthma and that bariatric surgery for morbid obesity is often followed by a decrease in the severity and extent of asthma symptoms (Dixon et al., 2011; Sutherland, 2014).

The overall impact of obesity on lung function appears to be multifactorial and related to both mechanical and inflammatory aspects of the excessive adipose tissue (Sutherland, 2014; Dixon and Peters, 2018). Obese individuals display infiltration of adipose tissue by activated macrophages. Adipocytes and macrophages are both responsible for the secretion of inflammatory mediators in proportion to the tissue mass, referred to collectively as adipokines (Trayhurn and Wood, 2004; Cancello et al., 2005). Most of them (such as tumor necrosis factor (TNF)-α, interleukin (IL)-6, IL-1β, leptin, and resistin) are pro-inflammatory and can alter lung function in a variety of ways (e.g. via the activation of vascular endothelial cells, airway fibroblasts, airway smooth muscle cells and airway epithelial cells) (Ouchi et al., 2011; Lau et al., 2017). On the contrary, levels of a few adipokines (including adiponectin (APN)) are low in obese individuals (Arita et al., 1999); these adipokines generally exhibit anti-inflammatory properties and protect against obesity-related diseases (Ouchi et al., 2011). Overall, an imbalance in the production of pro- and anti-inflammatory adipokines in the obese individual leads to complications—including cardiovascular diseases, infectious susceptibility, or even asthma and other inflammatory pulmonary complications (Ouchi et al., 2011; Zheng et al., 2016).

The airway epithelium acts as a frontline defense against foreign agents like allergens, viruses, and pollutants. It constitutes both a physical barrier (through the mucociliary apparatus) and an immunologic barrier. The bronchial epithelium's ability to control the balance between inhibitory and activating signals is essential for orchestrating appropriate inflammatory and immune responses and resolving inflammation during tissue repair (Loxham and Davies, 2017). When influenza viruses infect the airway epithelium, they trigger crucial innate and adaptive immune antiviral responses. The interaction between respiratory viruses and airway epithelial cells results in production of antiviral substances, cytokines, and chemokines, which recruit inflammatory cells and influence the innate and adaptive immunity(Vareille et al., 2011; Herold et al., 2015; Veerapandian et al., 2018). With respect to asthma, the evidence for epithelial dysregulation is compelling; and the consensus is that the initiating trigger occurs at the bronchial epithelium (Vareille et al., 2011; Herold et al., 2015).

Given the epithelium's major role in both respiratory tract viral infections and asthma, and the accentuating role of obesity in these diseases, the objectives of the present study were to (i) investigate the effects of adipokines (adiponectin (APN), leptin, chemerin, and visfatin) on the production of cytokines by unstimulated or TNF-α- or poly(I:C)-activated primary human bronchial epithelial cells (hBECs), (ii) study the expression of the corresponding adipokine receptors, and (iii) assess the production of the relevant adipokines by hBECs. We have chosen a set of cytokines (IL-6, CCL2, CCL5, CCL20, CXCL1, and CXCL8) reproducibly released by hBECs stimulated with TNF-α or Poly(I:C), and involved in the recruitment and/or activity of the neutrophils (CXCL8, CXCL1, and CXCL5), the eosinophils (CCL5), the monocytes/macrophages (CCL2), the myeloid dendritic cells (CCL20) and the lymphocytes T helper 1 (CCL5, CXCL10) (Vareille et al., 2011).

## Materials and Methods

### Materials

Antibiotics (penicillin, streptomycin, gentamycin, vancomycin, amphotericin, and ceftazidim), DMSO, L-glutamine, heat-inactivated fetal calf serum (FCS), protease (bovine pancreas) and HEPES were purchased from Sigma^®^ (St. Louis, MO, USA). Roswell Park Memorial Institute (RPMI) 1640 medium, Dulbecco's Modified Eagle's medium (DMEM) High Glucose medium, and bovine serum albumin were obtained from Eurobio Biotechnology^®^ (Les Ulis, France). Bronchial epithelial cell growth medium (BEGM, with growth factors and antibiotics) and Mycozap^®^ were purchased from Lonza^®^ (Basel, Switzerland). Trypsin 0.25% EDTA was obtained from Gibco^®^ (ThermoFisher Scientific, Waltham, MA, USA). Culture flasks for epithelial cell culture were from TPP^®^, and pre-coated culture flasks were from Corning^®^ (NY, USA). All the other cell culture plastics were from CML (Nemours, France).

Human recombinant TNF-α was bought from Bio-Techne^®^ (Lille, France). High-molecular-weight poly(I:C) was obtained from InvivoGen^®^ (Toulouse, France). Full-length human recombinant adiponectin (produced in *E. coli*) was purchased from Biovendor^®^ (Karasek, Czech Republic), and was solubilized in RPMI. Human recombinant leptin, visfatin and chemerin were also *E. coli*-derived. Leptin and chemerin were purchased from Bio-Techne^®^ (Lille, France), and visfatin was purchased from PeproTech^®^ (Neuilly-sur-Seine, France).

### Tissue Preparation

The use of resected lung tissue for *in vitro* experiments was approved by the local institutional review board (*Comité de Protection des Personnes Ile de France VIII*, Boulogne-Billancourt, France). Lung tissue samples were obtained from 22 patients (11 males and 11 females; 10 current smokers, 9 former smokers, and 3 non-smokers; mean ± standard deviation (SD) age: 63.4 ± 10.6 years; mean Forced Expiratory Volume in 1 second (FEV1) = 88.0 ± 27.3%; mean pack-years: 32.0 ± 19; mean FEV1/Forced Vital Capacity (FVC) ratio: 0.75 ± 0.16) undergoing surgical resection for lung carcinoma and who had not previously undergone chemotherapy (**Table S1** in supplemental data). Written patient's consent for the use of their pulmonary samples for research purpose was obtained prior operation.

Lung samples were isolated from macroscopically normal lung parenchyma obtained from sites distant from the tumor. To obtain bronchial rings, dissection was performed by starting at the main bronchus and working down to the 4^th^ or 5^th^ level of bronchial division. Bronchial segments were excised after the removal of adhering lung parenchyma and connective tissue. They were then either immediately crushed for RNA extraction or cultured for 24 h in culture plates containing RPMI supplemented with L-glutamine, 10% FCS and antibiotics, prior to recovery of the supernatant for the adiponectin assay.

### Isolation and Culture of Human Bronchial Epithelial Cells

The proximal segmental bronchi were placed in DMEM High Glucose supplemented with L-glutamine, 10% FCS, HEPES, and antibiotics/antifungal drugs (penicillin, streptomycin, gentamycin, vancomycin, amphotericin, ceftazidime, and mycozap), and then stored at +4°C for at least three days for bacterial decontamination. Bronchial mucus was removed, and the bronchus was cut lengthwise before the addition of protease (10 mg.ml^-1^) overnight at +4°C. On the following day, the inside of the bronchus was gently scratched in order to detach cells into the culture medium; the latter was then centrifuged (room temperature (RT), 650 rpm, 5 min). The cell pellet was resuspended in 30 ml of supplemented high-glucose DMEM and placed in a culture flask for 2 h at 37°C (5% CO_2_). Only the supernatant was recovered, and placed in a pre-coated culture flask. On the following day and every other day thereafter, the culture medium was replaced (BEGM-DMEM: 15 ml high-glucose DMEM without antibiotics and 15 ml BEGM). When the cells were confluent (after ~7 days of culture), they were detached with trypsin, centrifuged (RT, 650 rpm, 5 min), and resuspended with BEGM-DMEM medium in a 24-well plate (1 ml per well; 50,000 to 100,000 cells by well).

### Treatment of Human Bronchial Epithelial Cells

When the cells were confluent, the 24-well plates were washed and 1 ml of fresh BEGM-DMEM medium was added to each well. Full-length human recombinant adiponectin (3, 10, 30 µg.ml^-1^ of culture medium), leptin (1, 10, 100, 1000 ng.ml^-1^), visfatin (50, 100, 250, 500 ng.ml^-1^) or chemerin (10, 100, 1000 ng.ml^-1^) was then added. After 1 h incubation with the adipokines, TNF-α (50 ng.ml^-1^) or poly(I:C) (10 µg.ml^-1^) was then added for a 24 h incubation period, cell culture supernatants were then collected and stored at -80°C for later analysis.

### Cytokine, Adiponectin, and LDH Assays

The concentrations of selected chemokines and cytokines in the hBEC supernatants were measured using ELISAs (Duoset Development System, Bio-Techne^®^, Lille, France), according to the manufacturer's instructions. The assays' limits of detection were 4 pg.ml^-1^ for CCL3, 8 pg.ml^-1^ for CCL2, 9 pg.ml^-1^ for IL-6, and 16 pg.ml^-1^ for CCL20, CXCL1, CCL5, and CXCL8.

The supernatant concentrations of APN in cultures of bronchial segments and hBECs were measured using the Quantikine Human Total Adiponectin Immunoassay (Bio-Techne^®^, Lille, France), with a limit of detection of 0.246 ng.ml^-1^. Cell viability was assessed by measuring LDH release with the CytoTox96^®^ Non-Radioactive Cytotoxicity Assay (Promega^®^, Madison, USA).

### Reverse Transcriptase – Quantitative Polymerase Chain Reaction (RT-qPCR) Assay

The RT-qPCR assays were conducted on hBECs or on bronchial segments crushed and homogenized in TRIzol^®^ reagent immediately after dissection, using a ball mill TissueLyser LT (Qiagen^®^, Courtaboeuf, France). The procedure for extracting total RNA from homogenates of human bronchi was adapted from that described by Chomczynski and Sacchi (Chomczynski and Sacchi, 1987). The amount of RNA extracted from hBECs or bronchi was estimated by spectrophotometry at 260 nm (Spectrophotometer Biowave DNA; Biochrom, Cambridge, UK), and its quality was measured with a microfluidic electrophoresis system (RNA Standard Sensitivity kits for Experion^®^, BioRad, Marnes-la-Coquette, France). After treatment with DNase (Life Technologies), 1 µg of total RNA was reverse-transcribed (SuperScript^®^ III First-Strand SuperMix Kit, Life Technologies). The resulting cDNA was then used for quantitative real-time PCR experiments with TaqMan^®^ chemistry (Life Technologies, ThermoFisher Scientific, Waltham, MA, USA). The cDNA was amplified using 20 ng of cDNA (Gene Expression Master Mix, Life Technologies) in a StepOnePlus thermocycler (Life Technologies, ThermoFisher Scientific, Waltham, MA, USA). The amplification conditions were as follows: initial denaturation at 95°C for 10 min, followed by 40 annealing/extension cycles (95°C for 15 s, then 60°C for 1 min). Fluorescence was measured after each cycle, and the threshold cycle (Ct) of the real-time PCR was defined as the point at which a fluorescence signal corresponding to the amplification of a PCR product was detectable. The reaction volume was set to 10 µl. Specific TaqMan^®^ arrays based on predesigned reagents (*AdipoR1*: Hs01114951_m1; *AdipoR2*: Hs00226105_m1, Life Technologies, ThermoFisher Scientific, Waltham, MA, USA) were used to analyze AdipoR1 and AdipoR2 transcripts. The housekeeping gene coding for hypoxanthine phosphoribosyltransferase (*HPRT1*: Hs99999909_m1) was used for signal normalization.

### Immunostaining of the Adiponectin Receptors (AdipoR1 and AdipoR2) on Human Bronchial Epithelial Cells

Primary human epithelial cells were fixed in methanol 80% on coverslip. After a 30 min-long saturation with phosphate buffered saline containing 1% bovine serum albumin, immunostaining was realized using primary antibodies targeting AdipoR1 (monoclonal rabbit Ig, dilution 1/100 - Enzo Life Sciences, Villeurbanne, France) or AdipoR2 (monoclonal rabbit Ig, dilution 1/30 – Abcepta, San Diego, USA). After 1 h of incubation, a secondary antibody (donkey monoclonal anti rabbit Ig coupled with fluorescent probe Alexa Fluor 488 (green- Invitrogen, Life Technologies Corp., Oregon, USA) was added. DAPI (Invitrogen, Life Technologies Corp., Oregon, USA) was used to stain the cell nuclear (blue). The images were processed using a Leica confocal microscope (X40).

### Statistical Analysis

Assay data were expressed as the mean ± standard error of the mean (SEM) per 10^6^ epithelial cells, mean ± SEM per 100 mg lung tissue or mean ± SEM per ml, as appropriate. The relative expression of mRNAs was calculated according to the 2^(-ΔCt)^ method (Livak and Schmittgen, 2001). In all the series of experiments, “n” corresponds to the number of donors from whom hBECs were isolated. The statistical analyses involved a one-way repeated-measures analysis of variance (Friedman's test), followed by Bonferroni's or Dunn's post-tests or t-tests, as appropriate. All analyses were performed using GraphPad Prism^®^ software (version 5.01, GraphPad Software Inc., San Diego, CA, USA). The threshold for statistical significance was set to *p* < 0.05.

## Results

### Effects of Adiponectin on Cytokine Production by Unstimulated Human Bronchial Epithelial Cells

**Figure 1:**
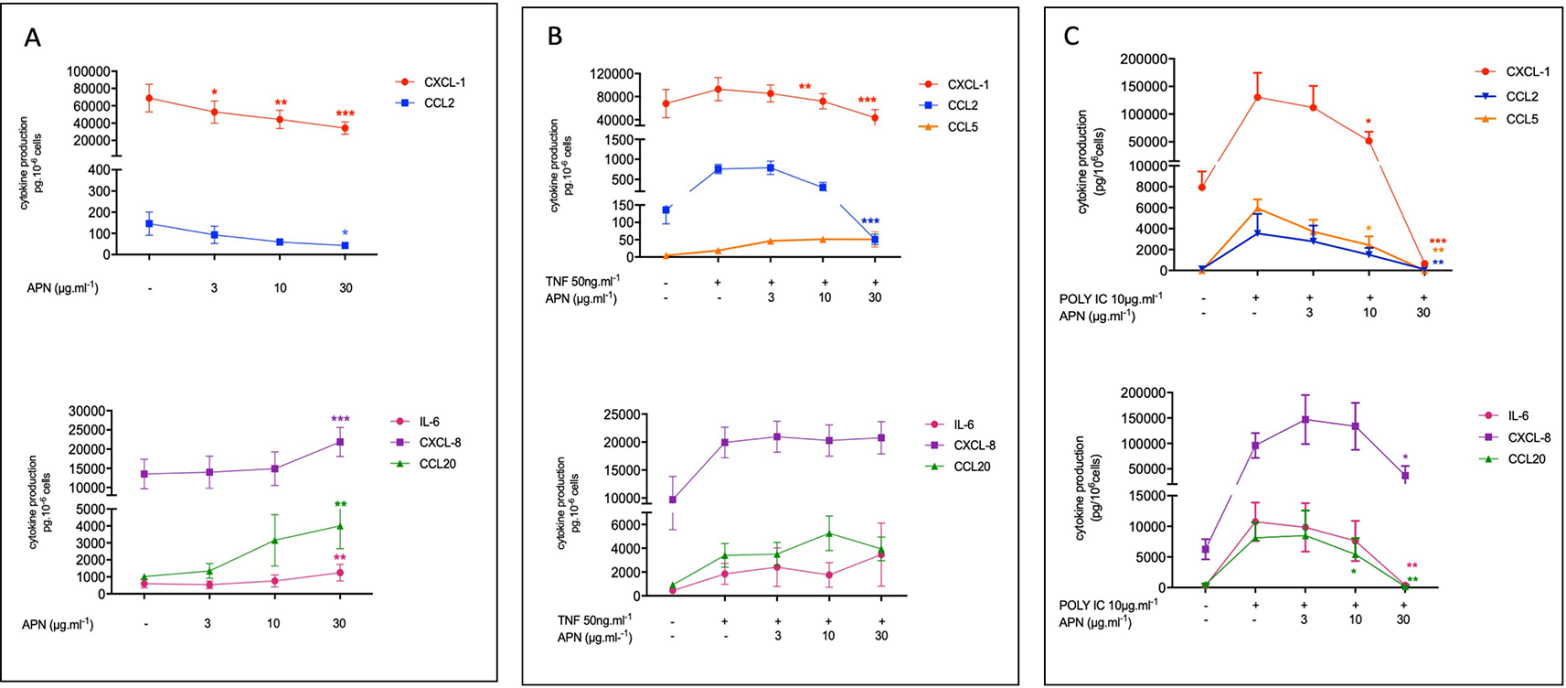
Amounts of cytokine in the supernatants of human bronchial epithelial cells treated with adiponectin in the absence **(A)** or presence of TNF-α **(B)** or Poly(I:C) **(C)**. Bronchial epithelial cells were incubated with adiponectin (APN) (3, 10, 30 µg.ml^-1^) in the absence of any stimulation **(A)** or in the presence of TNF-α (50 ng.ml^-1^) **(B)** or Poly(I:C) (10 µg.ml^-1^) **(C)**. Cell culture supernatants were collected after 24 h of incubation and analyzed using ELISAs. Amounts (pg.10^-6^cells) are quoted as the mean ± SEM of paired experiments performed on hBECs isolated from 5 to 9 independent donors. * p < 0.05; ** p< 0.01; *** p < 0.001.

Adiponectin (3, 10 or 30 µg.ml^-1^) exerted contrasting effects on the basal production of cytokines by hBECs; it was associated with lower production of CXCL1 and CCL2 in a concentration-dependent manner. At the highest concentration of APN (30 µg.ml^-1^), the production of CXCL1 and CCL2 was 2-fold and 3.75-fold lower than in control experiments, respectively. In contrast, adiponectin was associated with a greater production of CCL20, CXCL8 and IL-6 reaching 3.9-fold, 1.62-fold, and 2.1-fold, respectively, at the highest concentration of adiponectin (**Figure 1A**; see also **Table S2** in supplemental data). Adiponectin exerted no effect on the very low basal levels of CCL5 production.

### Effects of Adiponectin on the TNF-α- and poly(I:C)-Induced Productions of Cytokines by Human Bronchial Epithelial Cells

The exposure of hBECs to TNF-α led to a 1.3-fold relative increase in the production of CXCL1 (compared with production by non-exposed cells); this increase was 5.9-fold for CCL2, 1.5-fold for CXCL8, 3.9-fold for IL-6, 3.6-fold for CCL20 and 5.1-fold for CCL5. Adiponectin inhibited the TNF-α-induced production of CXCL1 and CCL2 but did not significantly affect the TNF-α-induced production of IL-6, CXCL8, CCL5, or CCL20 (**Figure 1B**; see also **Table S2** in supplemental data).

The exposure of hBECs to poly(I:C) led to a 16.4-fold relative increase in the production of CXCL1 (compared with non-exposed cells); this increase was 27-fold for CCL2, 15.4-fold for CXCL8, 38.2-fold for IL-6, 21.4-fold for CCL20, and 697-fold for CCL5. Adiponectin inhibited the poly(I:C)-induced production of all assayed cytokines: CXCL1, CCL2, CCL5, CCL20, IL6, and (albeit to a lesser extent) CXCL8 (**Figure 1C**; see also **Table S2** in supplemental data).

The incubation with adiponectin alone (30 µg.ml^-1^) or in presence of poly(I:C) or TNF-α was associated with a very weak increase in LDH release by the epithelial cells, thus demonstrating the absence of cytotoxicity (**Table S3** supplemental data).

### Effects of Leptin, Chemerin, and Visfatin on Cytokine Production by Human Bronchial Epithelial Cells

**Figure 2:**
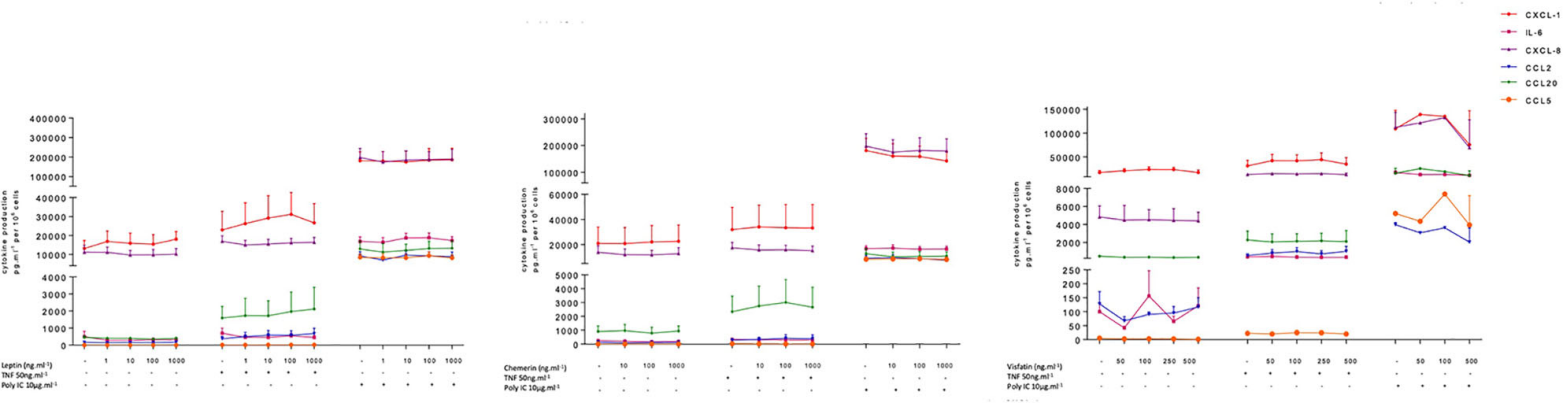
Amounts of cytokine in the supernatants of human bronchial epithelial cells treated with leptin, visfatin or chemerin in the absence or presence of TNF-α or poly(I:C).Bronchial epithelial cells were incubated with leptin (1, 10, 100, 1000 ng.ml^-1^), chemerin (10, 100, 500 ng.ml^-1^) or visfatin (50, 100, 250, 1000 ng.ml^-1^) in the absence of any stimulation **(A)** or in the presence of TNF-α (50 ng.ml^-1^) or poly(I:C) (10 µg.ml^-1^). Cell culture supernatants were collected after 24 h of incubation and analyzed using ELISAs. Amounts (pg.10^-6^cells) are quoted as the mean ± SEM of paired experiments performed on hBECs isolated from 4 to 9 independent donors (excepted for visfatin in the presence of Poly(I:C), n = 2).

The hBECs were also treated with a range of concentrations of leptin, visfatin and chemerin. These three adipokines did not have significant effects on the production of IL-6, CCL2, CCL5, CCL20, CXCL1, or CXCL8 by unstimulated or stimulated hBECs (**Figure 2** and **Table S4** in supplemental data). None of these three adipokines affected the viability of the hBECs after 24 h of exposure (data not shown).

### Expression of AdipoR1 and AdipoR2 by Primary Human Bronchial Epithelial Cells and Bronchial Explants

**Figure 3:**
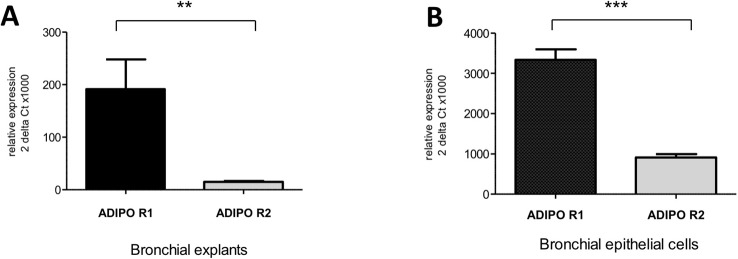
Expression of the adiponectin receptors type 1 and 2 in human bronchi **(A)** and primary bronchial epithelial cells **(B)**. Adiponectin receptor expression was assessed using qRT-PCR assays. The results are quoted as the mean ± SEM of paired experiments performed on hBECs isolated from 4 to 6 independent donors. ** p< 0.01; *** p < 0.001.

**Figure 4:**
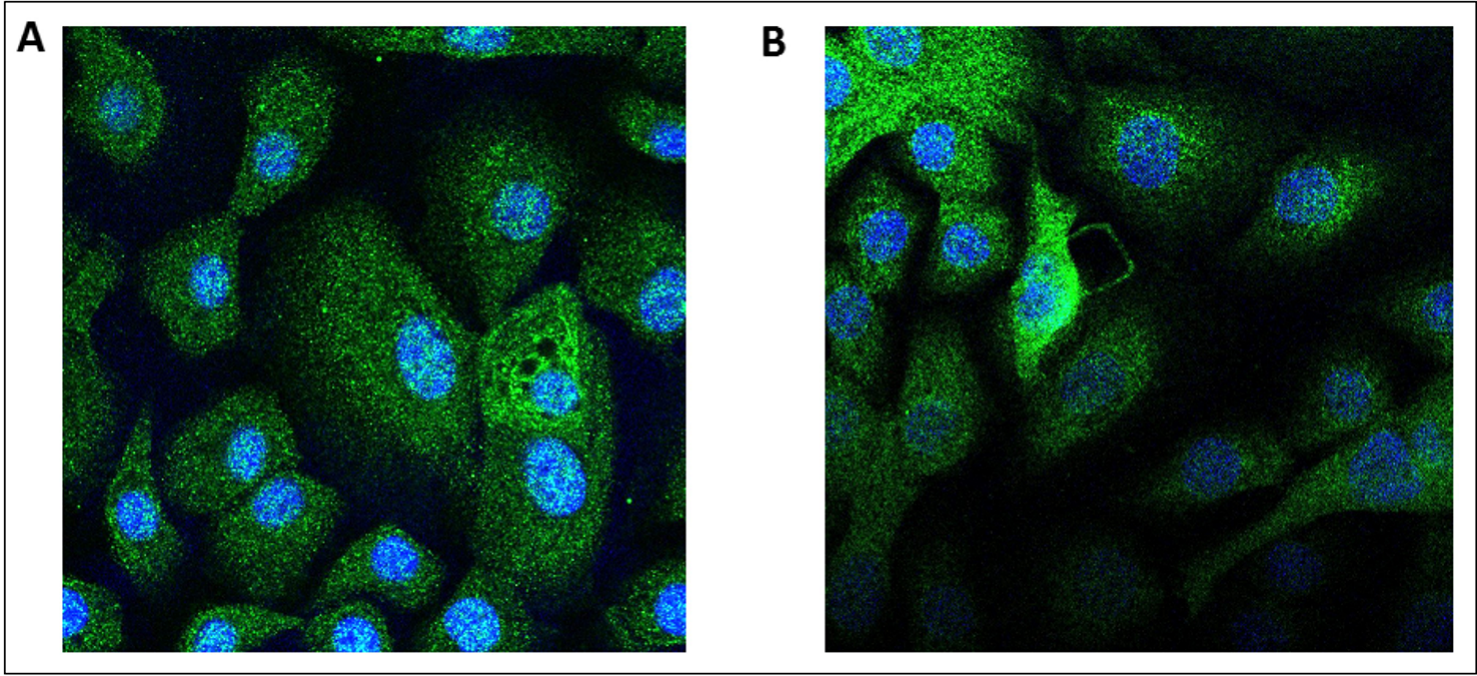
Immunostaining of the adiponectin receptors 1 and 2 in human primary bronchial epithelial cells. Human bronchial epithelial cells were immunostained with either an anti-AdipoR1 **(A)** or anti-AdipoR2 **(B)** antibodies. In absence of the primary antibodies, the secondary antibody coupled with a fluorescent probe do not stain the cells.

Transcripts of both AdipoRs subtypes were detected in the RNA extracted from hBECs and from bronchial explants (**Figure 3**). The expression of AdipoR1 was about 4-fold higher than that of AdipoR2 in hBECs, and 10-fold higher in explants. We have also compared the expression of the AdipoRs in paired bronchial rings with an intact epithelium and without epithelium after gentle scratching. The results suggest that the epithelium accounts for about 50% of the AdipoR expression in isolated human bronchial rings (**Figure S1**). The expression of the two types of AdipoRs on hBECs was also detected by immunostaining and confocal microscopy (**Figure 4**).

### Production of APN by Primary Human Bronchial Epithelial Cells and Bronchial Explants

Adiponectin was found at a mean ± SEM concentration of 28.8 ± 3 ng.ml^-1^ per 100 mg in the supernatants of human bronchial explants (n = 12) but was not detected in the supernatants of hBECs (n = 7). After adjustment for the volumes of the culture medium (2.5 ml) and the bronchial explant (100 mg ≈ 0.1 ml), this corresponds to an estimated tissue concentration of 0.72 µg.g^-1^.

## Discussion

The present study of the effects of four adipokines on hBECs highlighted (i) APN's contrasting effects on basal cytokine production, (ii) APN's inhibitory effects on TNF-α- and poly(I:C)-induced cytokine production, and (iii) AdipoRs expression by hBECs. Furthermore, we found that APN was produced by bronchial explants but not by isolated hBECs.

The few reports on the effect of adipokines on basal cytokine release from lung epithelial cells have generated divergent results. In a study of the A549 human alveolar basal epithelial cell line (a type II pulmonary epithelial cell model), Miller et al. (Miller et al., 2009) demonstrated that the C-terminal globular fragment of APN (1 µg.ml^-1^ for 24 h) significantly induced CXCL-8 release. However, in Nigro et al.'s study (Nigro et al., 2013) of the same cell line, incubation with full-length APN (5 or 50 µg.ml^-1^ for 14 h) did not influence the mRNA expression of CXCL-8, CCL-2, and IL-6. Furthermore, Chen et al. reported that full-length APN upregulated cytosolic phospholipase A2 and cyclooxygenase-2 in the A549 cell line (Chen et al., 2016). The A549 cell line is a hypotriploid human cell line initiated by the explant culture of lung carcinomatous tissue. To the best of our knowledge, our present study is the first to have shown that APN differentially regulates basal cytokine production in primary hBECs. Adiponectin upregulated the basal production of CCL20, CXCL8, and IL-6 (without enhancing their production in TNF-α- or poly(I:C)-stimulated cells), downregulated the basal production of CXCL1 and CCL2, and did not influence a very low basal production of CCL5.

Furthermore, there are few data on the adipokines' effects on cytokine production by stimulated human airway epithelial cells. In the A549 cell line, APN (5 or 50 µg.ml^-1^ for 14 h) did not influence the TNF-α- and IL-1β-induced increases in the mRNA expression of CXCL8, CCL2, and IL-6 but did enhance IL-10 mRNA expression (Nigro et al., 2013). On experiments in hBECs, Cheng et al. (Cheng et al., 2016) showed that both the full-length and globular forms of APN (5 or 20 µg.ml^-1^) inhibited the expression of TNF-α and CXCL-8 induced by cigarette smoke extract in a concentration-dependent manner. Again, to the best of our knowledge, the present study on primary hBECs is the first to show that APN downregulated the TNF-α-induced production of CXCL1 and CCL2, and the poly(I:C)-induced production of IL-6, CCL2, CCL5, CCL20, CXCL1, and CXCL8. We chose to use TNF-α to stimulate the hBECs because it reportedly increases cytokine production (IL-1β, TNF-α IL-6, CXCL-8, and TSLP) in this cell type, and is known to promote epithelial barrier dysfunction (Hardyman et al., 2013). Poly(I:C), similar to double-stranded RNA in its structure, was used to simulate viral interaction, and reportedly stimulates the production of high levels of various cytokines and chemokines by hBECs (Royer et al., 2017). In contrast, LPS (a Toll-like receptor 4 agonist) was not associated with an increase in cytokine production by hBECs [(Royer et al., 2017) and data not shown]. The inhibitory effect of APN on the Poly(I:C)-induced production of CCL2, CCL5, CCL20, CXCL1, and CXCL8 might explain why viral infections [and particularly infections by the H1N1 influenza virus (Salvator et al., 2011)] are particularly severe in obese patients since the concentrations of APN are abnormally low in obese individuals (Ouchi et al., 2011). Indeed, these five chemokines are at least in part involved in the recruitment and/or activity of the neutrophils (CXCL8, CXCL5), the eosinophils (CCL5), the monocytes/macrophages (CCL2), the myeloid dendritic cells (CCL20) and the lymphocytes T helper 1 (CCL5, CXCL10) (Vareille et al., 2011). These cell types are involved in the innate and adaptive immune responses to viral infections (Vareille et al., 2011). However, we did not measured CCL3, a chemokine involved in the recruitment and activity of NK cells. This latter cell type are also important in early antiviral responses and in the antigen-independent activation of antigen-presenting cells (Vareille et al., 2011). Further work will be necessary to explore the molecular mechanisms involved in the inhibitory effect of APN on the Poly(I:C)-induced production of inflammatory cytokines. In addition to regulating cytokine production, APN might protect alveolar epithelial cells (A549 cells) by counteracting the cytotoxic effects of TNF-α and IL-1β and by regulating cell viability (Nigro et al., 2013). It has been reported that APN has a weak effect on A549 cell viability (according to a tetrazolium MTT assay) (Nigro et al., 2013) but we did not find any impact of APN on hBEC viability. Adiponectin might also protect bronchial epithelial cells by regulating proliferation, wound repair, and apoptosis (Zhu et al., 2013).

In studies with A549 cells, AdipoR1 (but not AdipoR2) was strongly expressed after stimulation with TNF-α (as assessed with RT-PCR, immunohistochemistry, and Western Blot), whereas unstimulated A549 cells did not express AdipoR1 (Miller et al., 2009). In two different studies, the two subtypes of AdipoRs were found to be expressed at the mRNA and protein levels in A549 cells and in two other human lung cell lines (SW-1573 and Calu-3) (Chen et al., 2016; Cheng et al., 2016). However, an immunohistochemistry study of lung sections from patients with COPD demonstrated that airway epithelial cells expressed significant levels of AdipoR1 but not of AdipoR2 (Miller et al., 2009). The immortalized human bronchial epithelial cell line (16HBE14o) expressed both AdipoRs but AdipoR1 indicated higher expression than AdipoR2 (Zhu et al., 2013). Similarly, our present results demonstrated that AdipoR1 was expressed more strongly than AdipoR2 in hBECs. Adiponectin binds to T-cadherin, in addition to the AdipoRs. However, T-cadherin does not possess the transmembrane or intracellular domain generally required for signaling, which hinders the protein's function as an adiponectin receptor. The APN/T-cadherin complex has been shown to protect against cellular damage through exosome biogenesis and secretion (Obata et al., 2018). An assessment of the putative function of APN bound to T-cadherin on hBECs was beyond the scope of the present study.

We also showed that APN was produced by human bronchial explants but not by the constituent hBECs. In contrast, an immunohistochemistry study of lung sections suggested that distal airway epithelial cells (but not alveolar cells) from patients with COPD (but not from controls and non-COPD smoker patients) expressed significant levels of APN (Miller et al., 2009). The apparent discrepancy between our results with isolated hBECs and the results of the latter immunohistochemistry study (Miller et al., 2009) might be due to differences between the study populations [i.e., mostly non-COPD smokers or former smokers in the present study versus COPD patients in Miller et al.'s study (Miller et al., 2009)] or differences in the methods [ELISAs with hBECs isolated from proximal bronchi and cultured for ~7 days in submerged conditions *versus* immunostaining of the in situ distal airway epithelium (Miller et al., 2009)]. After taking account of dilution in the culture medium, the estimated APN concentration in lung tissue is close to the range found in the blood (between 5 to 20 µg.ml^-1^) and close to the concentrations of recombinant APN found to modulate cytokine production by hBECs (our present data). Identification of the cell types that produced APN was beyond the scope of the present study. However, our results demonstrate that the APN produced locally by the lung parenchyma and bronchus may have paracrine effects on epithelial cells.

Adiponectin is not the only adipokine synthetized in the lungs. Leptin is reportedly synthetized by bronchial epithelial cells and alveolar type II pneumocytes (Bruno et al., 2009; Vernooy et al., 2009), and chemerin is expressed (as mRNA transcripts and as protein) by the bronchiolar epithelium in mice (Luangsay et al., 2009). In the present study, neither leptin, visfatin nor chemerin modulated the production of cytokines by hBECs. Similarly, it has been reported that leptin had no effect on hBECs proliferation, wound repair, and apoptosis (Zhu et al., 2013). Visfatin (also referred to as nicotinamide phosphoribosyltransferase and pre-B cell colony-enhancing factor) is reportedly a pro-inflammatory factor for monocytes and macrophages (Travelli et al., 2018). In A549 cells, visfatin stimulated the expression of CXCL8 and IL-16 via its non-enzymatic activity (Liu et al., 2009). The transfection of A549 and human alveolar epithelial cells with visfatin was also associated with enhanced CXCL8 and TNF-α expression and production (Li et al., 2008; Liu et al., 2009), whereas the inhibition of visfatin's enzyme activity was associated with a relative reduction in the production of CXCL8 caused by hypoxia-reoxygenation of A549 cells (Wu et al., 2017). However, in contrast with these literature results for the A549 epithelial alveolar cell line, we were unable to detect a modulatory effect of visfatin on cytokine production by hBECs. Furthermore, it has been suggested that chemerin can regulate lung inflammation; chemerin receptor (ChemR23) knock-out mice produced lower levels of inflammatory cytokines (TNF, CCL2, CXCL-1, and CCL20) after a respiratory challenge with cigarette smoke or a virus (Demoor et al., 2011; Bondue et al., 2011). Again, we did not observe a modulatory effect of chemerin on hBECs.

In conclusion, our present results suggest that of the four adipokines tested, only APN may modulate the bronchial epithelium's inflammatory responses in general and responses to viral infection in particular. We evidenced (i) AdipoR1 and AdipoR2 transcript expression by hBECs, and (ii) the production of APN by bronchial explant—suggesting that APN can modulate cytokine production by bronchial epithelial cells in a paracrine manner. Furthermore, our results suggest that the abnormally low APN concentration associated with obesity may contribute to susceptibility to viral lung infections and the severity of these infections in obese individuals.

## Author’s Note

These results have been presented in part at the Annual Meeting of French Society of Pharmacology and Therapeutics and INSERM Clinical Research Centers (CIC) Meeting, June 2019, Lyon, France.

## Data Availability Statement

The datasets generated for this study are available on request to the corresponding author.

## Author Contributions

HS designed experimental plan, realized the experiments, analyzed the data and wrote the manuscript. SG designed experimental plan. EN designed experimental plan and realized the experiments. MB realized the experiments. CF realized the experiments. L-JC analyzed the data. PD designed experimental plan, analyzed the data and wrote the manuscript. All authors read and approved the final manuscript.

## Conflict of Interest

The authors declare that the research was conducted in the absence of any commercial or financial relationships that could be construed as a potential conflict of interest.

## Abbreviations

APN, adiponectin; AdipoR, adiponectin receptor; BEGM, Bronchial epithelial cell growth medium; BMI, body mass index; DMEM, Dulbecco's Modified Eagle's medium; DMSO, Diméthylsulfoxyde; ELISA, Enzyme-linked immunosorbent assay; FCS, Heat-inactivated fetal calf serum FEV1, Forced Expiratory Volume in 1 second; FVC, Forced Vital Capacity; hBEC, primary human bronchial epithelial cell; IL, interleukin; LPS, lipopolysaccharide; RPMI, Roswell Park Memorial Institute; RNA, Ribonucleic Acide; RT-qPCR, Reverse transcriptase – quantitative polymerase chain reaction; TNF-α, Tumor necrosis factor-α.

## References

[B1] Arita Y. Kihara S. Ouchi N. Takahashi M. Maeda K. Miyagawa J. (1999). Paradoxical decrease of an adipose-specific protein, adiponectin, in obesity. Biochem. Biophys. Res. Commun. 257, 79−83. 10.1006/bbrc.1999.0255 10092513

[B2] Bondue B. Vosters O. de Nadai P. Glineur S. De Henau O. Luangsay S. (2011). ChemR23 dampens lung inflammation and enhances anti-viral immunity in a mouse model of acute viral pneumonia. PLoS Pathog. 7 (11), e1002358. 10.1371/journal.ppat.1002358 22072972 PMC3207933

[B3] Boulet L.-P. Turcotte H. Martin J. Poirier P. (2012). Effect of bariatric surgery on airway response and lung function in obese subjects with asthma. Respir. Med. 106, 651–660. 10.1016/j.rmed.2011.12.012 22326605

[B4] Bruno A. Pace E. Chanez P. Gras D. Vachier I. Chiappara G. (2009). Leptin and leptin receptor expression in asthma. J. Allergy Clin. Immunol. 124, 230−7, 237.e1–4. 10.1016/j.jaci.2009.04.032 19539983

[B5] Cancello R. Henegar C. Viguerie N. Taleb S. Poitou C. Rouault C. (2005). Reduction of macrophage infiltration and chemoattractant gene expression changes in white adipose tissue of morbidly obese subjects after surgery-induced weight loss. Diabetes 54, 2277−86. 10.2337/diabetes.54.8.2277 16046292

[B6] Chen H.-M. Yang C.-M. Chang J.-F. Wu C.-S. Sia K.-C. Lin W.-N. (2016). AdipoR-increased intracellular ROS promotes cPLA2 and COX-2 expressions via activation of PKC and p300 in adiponectin-stimulated human alveolar type II cells. Am. J. Physiol. Lung Cell Mol. Physiol. 311, L255–L269. 10.1152/ajplung.00218.2015 27288489

[B7] Cheng M.-Y. Liu H. Zhang T.-M. Xu J.-Y. (2016). Different forms of adiponectin reduce the apoptotic and damaging effect of cigarette smoke extract on human bronchial epithelial cells. Exp. Ther. Med. 12, 4168−74. 10.3892/etm.2016.3872 28105143 PMC5228410

[B8] Chomczynski P. Sacchi N. (1987). Single-step method of RNA isolation by acid guanidinium thiocyanate-phenol-chloroform extraction. Anal. Biochem. 162, 156−9. 10.1016/0003-2697(87)90021-2 2440339

[B9] Demoor T. Bracke K. R. Dupont L. L. Plantinga M. Bondue B. Roy M.-O. (2011). The role of ChemR23 in the induction and resolution of cigarette smoke-induced inflammation. J. Immunol.Baltim. Md 1950 186, 5457−67. 10.4049/jimmunol.1003862 21430224

[B10] Dixon A. E. Peters U. (2018). The effect of obesity on lung function. Expert Rev. Respir. Med. 12, 755–767. 10.1080/17476348.2018.1506331 30056777 PMC6311385

[B11] Dixon A. E. Pratley R. E. Forgione P. M. Kaminsky D. A. Whittaker-Leclair L. A. Griffes L. A. (2011). Effects of obesity and bariatric surgery on airway hyperresponsiveness, asthma control, and inflammation. J. Allergy Clin. Immunol. 128, 508–515.e1-2. 10.1016/j.jaci.2011.06.009 21782230 PMC3164923

[B12] GBD 2015 Obesity Collaborators Afshin A. Forouzanfar M. H. Reitsma M. B. Sur P. Estep K. (2017). Health effects of overweight and obesity in 195 countries over 25 years. N. Engl. J. Med. 377, 13–27. 10.1056/NEJMoa1614362 28604169 PMC5477817

[B13] Hardyman M. A. Wilkinson E. Martin E. Jayasekera N. P. Blume C. Swindle E. J. (2013). TNF-α-mediated bronchial barrier disruption and regulation by src-family kinase activation. J. Allergy Clin. Immunol. 132, 665–675.e8. 10.1016/j.jaci.2013.03.005 23632299

[B14] Herold S. Becker C. Ridge K. M. Budinger G. R. S. (2015). Influenza virus-induced lung injury: pathogenesis and implications for treatment. Eur. Respir. J. 45, 1463−78. 10.1183/09031936.00186214 25792631

[B15] Lau W. B. Ohashi K. Wang Y. Ogawa H. Murohara T. Ma X.-L. (2017). Role of Adipokines in cardiovascular disease. Circ. J. Off. J. Jpn. Circ. Soc 81, 920−8. 10.1253/circj.CJ-17-0458 28603178

[B16] Li H. Liu P. Cepeda J. Fang D. Easley R. B. Simon B. A. (2008). Augmentation of pulmonary epithelial cell IL-8 expression and permeability by Pre-B-cell colony enhancing factor. J. Inflammation Lond. Engl. 5, 15. 10.1186/1476-9255-5-15 PMC2559829 18808711

[B17] Liu P. Li H. Cepeda J. Xia Y. Kempf J. A. Ye H. (2009). Regulation of inflammatory cytokine expression in pulmonary epithelial cells by pre-B-cell colony-enhancing factor *via a* nonenzymatic and AP-1-dependent mechanism. J. Biol. Chem. 284, 27344−51. 10.1074/jbc.M109.002519 19654329 PMC2785662

[B18] Liu P. Li H. Cepeda J. Zhang L. Q. Cui X. Garcia J. G. N. (2009). Critical role of PBEF expression in pulmonary cell inflammation and permeability. Cell Biol. Int. 33, 19−30. 10.1016/j.cellbi.2008.10.015 18996492 PMC3732657

[B19] Livak K. J. Schmittgen T. D. (2001). Analysis of relative gene expression data using real-time quantitative PCR and the 2(-Delta Delta C(T)) Method. Methods San Diego Calif. Methods 25 (4), 402–408. 10.1006/meth.2001.1262 11846609

[B20] Loxham M. Davies D. E. (2017). Phenotypic and genetic aspects of epithelial barrier function in asthmatic patients. J. Allergy Clin. Immunol. 139, 1736−51. 10.1016/j.jaci.2017.04.005 28583446 PMC5457128

[B21] Luangsay S. Wittamer V. Bondue B. De Henau O. Rouger L. Brait M. (2009). Mouse ChemR23 is expressed in dendritic cell subsets and macrophages, and mediates an anti-inflammatory activity of chemerin in a lung disease model. J. Immunol. Baltim. Md 1950 183, 6489−99. 10.4049/jimmunol.0901037 19841182

[B22] Maccioni L. Weber S. Elgizouli M. Stoehlker A.-S. Geist I. Peter H.-H. (2018). Obesity and risk of respiratory tract infections: results of an infection-diary based cohort study. BMC Public Health 18, 271. 10.1186/s12889-018-5172-8 29458350 PMC5819164

[B23] Miller M. Cho J. Y. Pham A. Ramsdell J. Broide D. H. (2009). Adiponectin and functional adiponectin receptor 1 are expressed by airway epithelial cells in chronic obstructive pulmonary disease. J. Immunol. Baltim. Md 1950 182, 684−91. 10.4049/jimmunol.182.1.684 19109202

[B24] Morgan O. W. Bramley A. Fowlkes A. Freedman D. S. Taylor T. H. Gargiullo P. (2010). Morbid obesity as a risk factor for hospitalization and death due to 2009 pandemic influenza A(H1N1) disease. PLoS One 5 (3), e9694. 10.1371/journal.pone.0009694 20300571 PMC2837749

[B25] NCD Risk Factor Collaboration (NCD-RisC) (2017). Worldwide trends in body-mass index, underweight, overweight, and obesity from 1975 to 2016: a pooled analysis of 2416 population-based measurement studies in 128·9 million children, adolescents, and adults. Lancet Lond. Engl. 390, 2627–2642. 10.1016/S0140-6736(17)32129-3 PMC5735219 29029897

[B26] Nigro E. Scudiero O. Sarnataro D. Mazzarella G. Sofia M. Bianco A. (2013). Adiponectin affects lung epithelial A549 cell viability counteracting TNFα and IL-1ß toxicity through AdipoR1. Int. J. Biochem. Cell Biol. 45, 1145−53. 10.1016/j.biocel.2013.03.003 23500159

[B27] Obata Y. Kita S. Koyama Y. Fukuda S. Takeda H. Takahashi M. (2018). Adiponectin/T-cadherin system enhances exosome biogenesis and decreases cellular ceramides by exosomal release. JCI Insight 19;3 (8), 99680. 10.1172/jci.insight.99680 PMC5931116 29669945

[B28] Ouchi N. Parker J. L. Lugus J. J. Walsh K. (2011). Adipokines in inflammation and metabolic disease. Nat. Rev. Immunol. 11, 85−97. 10.1038/nri2921 21252989 PMC3518031

[B29] Royer P.-J. Henrio K. Pain M. Loy J. Roux A. Tissot A. (2017). TLR3 promotes MMP-9 production in primary human airway epithelial cells through Wnt/β-catenin signaling. Respir. Res. 18, 208. 10.1186/s12931-017-0690-y 29237464 PMC5729411

[B30] Salvator H. Devillier P. Rivaud E. Catherinot E. Honderlick P. Couderc L.-J. (2011). [Obesity, poor prognostic factor in pandemic influenza A (H1N1) 2009: the role of adipokines in the modulation of respiratory defenses]. Rev. Pneumol. Clin. 67, 244−9. 10.1016/j.pneumo.2011.01.001 21920285

[B31] Sutherland E. R. (2014). Linking obesity and asthma. Ann. N. Y. Acad. Sci. 1311, 31−41. 10.1111/nyas.12357 24517401

[B32] Travelli C. Colombo G. Mola S. Genazzani A. A. Porta C. (2018). NAMPT: A pleiotropic modulator of monocytes and macrophages. Pharmacol. Res. 135, 25−36. 10.1016/j.phrs.2018.06.022 30031171

[B33] Trayhurn P. Wood I. S. (2004). Adipokines: inflammation and the pleiotropic role of white adipose tissue. Br. J. Nutr. 92, 347−55. 10.1079/BJN20041213 15469638

[B34] Van Kerkhove M. D. Vandemaele K. A. H. Shinde V. Jaramillo-Gutierrez G. Koukounari A. Donnelly C. A. (2011). Risk factors for severe outcomes following 2009 influenza A (H1N1) infection: a global pooled analysis. PLoS Med. 8 (7), e1001053. 10.1371/journal.pmed.1001053 21750667 PMC3130021

[B35] Vareille M. Kieninger E. Edwards M. R. Regamey N. (2011). The airway epithelium: soldier in the fight against respiratory viruses. Clin. Microbiol. Rev. 24, 210−29. 10.1128/CMR.00014-10 21233513 PMC3021210

[B36] Veerapandian R. Snyder J. D. Samarasinghe A. E. (2018). Influenza in asthmatics: for better or for worse? Front. Immunol. 9, 1843. 10.3389/fimmu.2018.01843 30147697 PMC6095982

[B37] Vernooy J. H. J. Drummen N. E. A. van Suylen R. J. Cloots R. H. E. Möller G. M. Bracke K. R. (2009). Enhanced pulmonary leptin expression in patients with severe COPD and asymptomatic smokers. Thorax 64, 26−32. 10.1136/thx.2007.085423 18835960

[B38] WHO (2017). 10 facts on obesity [Internet]. WHO. http://www.who.int/features/factfiles/obesity/en/.

[B39] Wu G.-C. Liao W.-I. Wu S.-Y. Pao H.-P. Tang S.-E. Li M.-H. (2017). Targeting of nicotinamide phosphoribosyltransferase enzymatic activity ameliorates lung damage induced by ischemia/reperfusion in rats. Respir. Res. 18, 71. 10.1186/s12931-017-0557-2 28438162 PMC5404693

[B40] Zheng H. Zhang X. Castillo E. F. Luo Y. Liu M. Yang X. O. (2016). Leptin enhances TH2 and ILC2 responses in allergic airway disease. J. Biol. Chem. 291, 22043−52. 10.1074/jbc.M116.743187 27566543 PMC5063987

[B41] Zhu X. L. Qin X. Q. Xiang Y. Tan Y. R. Qu X. P. Liu H. J. (2013). Adipokine adiponectin is a potential protector to human bronchial epithelial cell for regulating proliferation, wound repair and apoptosis: comparison with leptin and resistin. Peptides 40, 34−41. 10.1016/j.peptides.2012.11.017 23220445

