# Supplementary material for: Contrasting Effects of Adipokines on the Cytokine Production by Primary Human Bronchial Epithelial Cells: Inhibitory Effects of Adiponectin
Source: Front Pharmacol. 2020 Feb 18;11:56. doi: 10.3389/fphar.2020.00056 (PMC7040162; doi:10.3389/fphar.2020.00056)
Supplement: Supplementary file 1 [file DataSheet_1.docx]

**SUPPLEMENTAL DATA**

**Table S1. Demographic and clinical characteristics**

Lung samples were obtained from 22 patients (11 female, 11 male) operated for lung cancer with no previous systemic treatment (chemotherapy or thoracic radiotherapy)

|  | **N =22** |
| --- | --- |
| Sex (male/female) | 11 /11 |
| Age | 63.4 ± 10.6 |
| Smoking status  Current smoker  Former smoker  Never smoker | 10  9  3 |
| Pack-year (mean ±SD) | 32.0 ± 19 |
| BMI (mean ±SD) | 24.5 ± 4.9  BMI > 30: n=2 |
| FEV1 % theory (mean ±SD) | 88.0 ± 27.3 |
| FEV1/FVC (mean ±SD) | 0.75 ± 0.16  FEV1/FVC < 70%: n= 4 |

**Table S2. Amounts of cytokines in the supernatants of primary human bronchial epithelial cells treated with adiponectin in absence or presence of TNF-α or poly(I:C)**

Bronchial epithelial cells were incubated with adiponectin (APN) (3, 10, 30 µg.ml^-1^) in the absence of any stimulation (A) or in presence of TNF-α (50 ng.mL^-1^, added one hour after APN) (B) or poly(I:C) (10 µg.ml^-1^, added one hour after APN) (C). Cell culture supernatants were collected after 24h incubation and analyzed by ELISA. Results are shown as the means ± SEM of 4-6 independent experiments. * p<0.05; ** p< 0.01; *** p<0.001, versus “Basal”, “TNF alone” or “Poly(I:C) alone”.

**A.**

| ***Cytokine***  ***ng.10^-6^ cells*** | ***BASAL*** | ***APN 3*** | ***APN 10*** | ***APN 30*** |
| --- | --- | --- | --- | --- |
| ***CXCL1*** | 68.86 ± 16.0 | 52.81 ± 12,74 | 44.30 ± 10.50^**^ | 34.39 ± 7.11^***^ |
| ***CCL2*** | 0.15 ± 0.06 | 0.09 ± 0.04 | 0.06 ± 0.02 | 0.04 ± 0.01^*^ |
| ***IL-6*** | 0.60 ± 0.24 | 0.54 ± 0.23 | 0.76 ± 0,36 | 1.25 ± 0.49^**^ |
| ***CXCL8*** | 13.51 ± 3.87 | 14.02 ± 4.19 | 14.91 ± 4.36 | 21.86 ± 3.78^***^ |
| ***CCL20*** | 1.02 ± 0.2 | 1.35 ± 0.4 | 3.15 ± 1.5 | 4.00 ± 1.3 ^**^ |
| ***CCL5*** | 0.0044 ± 0.0018 | 0.0053 ±0.0026 | 0.0124±0.0044 | 0.0111± 0.0031 |

**B.**

| ***Cytokine***  ***ng.10^-6^ cells*** | ***BASAL*** | ***TNF alone*** | ***APN3***  ***+ TNF*** | ***APN 10***  ***+ TNF*** | ***APN30***  ***+ TNF*** |
| --- | --- | --- | --- | --- | --- |
| ***CXCL1*** | 61.23 ± 24.0 | 92.93 ± 20.28 | 85.59 ± 14.91 | 72.03 ± 13.40^**^ | 43.47 ± 13.96^***^ |
| ***CCL2*** | 0.14 ± 0.04 | 0.76 ± 0.12 | 0.79 ± 0.17 | 0.30 ± 0.13^**^ | 0.05 ± 0.02^***^ |
| ***IL-6*** | 0.44 ± 0.24 | 1.85 ± 0.89 | 2.41 ± 1.62 | 1.76 ± 1.04 | 3.47 ± 2.67 |
| ***CXCL8*** | 0.97 ± 4.14 | 19.93 ± 2.72 | 20.96 ± 2.76 | 20.26 ± 2.80 | 20.77 ± 2.89 |
| ***CCL20*** | 0.93 ± 0.18 | 3.34 ± 1.0 | 3.50 ± 1.0 | 5.25 ± 1.4 | 3.94 ± 1.0 |
| ***CCL5*** | 0.0044 ± 0.0018 | 0.0188 ± 0.0090 | 0.0466 ± 0.0051 | 0.0516±0.0083 | 0.0509 ± 0.0223 |

**C.**

| ***Cytokine***  ***ng.10^-6^ cells*** | ***BASAL*** | ***Poly(I:C) alone*** | ***APN3***  ***+ Poly (I:C)*** | ***APN 10***  ***+ Poly(I:C)*** | ***APN30***  ***+ Poly(I:C)*** |
| --- | --- | --- | --- | --- | --- |
| ***CXCL1*** | 79.39 ± 15.2 | 130.3 ± 99.2 | 111.9 ± 87.3 | 51.6 ± 37.5^*^ | 0.53 ± 0.5^***^ |
| ***CCL2*** | 0.13 ± 0.03 | 3.55 ± 4.52 | 2.78 ± 3.70 | 1.51 ± 1.60^**^ | 0.11 ± 0.11^**^ |
| ***IL-6*** | 0.28 ± 0.22 | 10.75 ± 7.05 | 9.84 ± 8.85 | 7.60 ± 7.36 | 0.36 ± 0.25^**^ |
| ***CXCL8*** | 6.24 ± 4.01 | 96.00 ± 59.5 | 146.97 ± 117.9 | 133.73 ± 113.1 | 36.76 ± 46.5^*^ |
| ***CCL20*** | 0.52 ± 042 | 11.09 ± 8.9 | 39.89 ± 8.87 | 8.07± 8.3^*^ | 0.25 ± 0.23^**^ |
| ***CCL5*** | 0.0085± 0.002 | 5.95 ± 1.9 | 3.72 ± 2.5 | 2.44 ±1.88^*^ | 0.01 ± 0.02^**^ |

**Table S3** Cytotoxicity after incubation with adiponectin (30 µg/ml^-1^), for 24 hr in culture medium. Cytotoxicity was measured using an LDH release assay. The data correspond to the LDH release (mean ± SEM of 4 to 5 independent experiments) corrected for baseline LDH release by cells incubated in culture medium alone and expressed as % of the maximum LDH release generated by using the provided lysis solution.

| Treatment | % of maximal  LDH release |
| --- | --- |
| Adiponectin (30 µg/ml^-1^) | 2.3 ±0.5 |
| TNF-α + adiponectin | 2.1 ± 0.7 |
| Poly(I:C) + adiponectin | 2.5 ±0.7 |

**Table S4.** Cytokines in the supernatants of human bronchial epithelial cells treated with leptin, chemerin or visfatin in absence (A) or presence of TNF-α (B) or Poly (I:C) (C).

Results are expressed in pg.10^6^cells and are shown as the mean ± SEM of 3-9 independent experiments. Only two experiments were performed with visfatin at the concentrations of 50, 100 and 250 ng.mL^-1^; no effect on the poly(I:C)-induced cytokine production was observed (data not shown).

**A**.

|  | | | | | | |
| --- | --- | --- | --- | --- | --- | --- |
|  | **Control (unstimulated)** | | | | | |
| Adipokines | IL-6 | CXCL-8 | CCL-2 | CXCL-1 | CCL20 | CCL5 |
| **Leptin (ng.mL^-1^)** | n=8 | n=9 | n=8 | n=7 | n=8 | n=8 |
| 0 | 495.6 ± 306.5 | 11179.9 ± 3594.9 | 154.1 ± 34.5 | 13118.8 ± 4262 | 455.7 ± 95.6 | 3.8 ± 2.1 |
| 1 | 280.7 ± 125.3 | 11071.8 ± 2789.7 | 132.8 ± 28.8 | 16869.1 ± 5502.3 | 400.6 ± 72.9 | 2.5 ± 1.0 |
| 10 | 289.2 ± 123.2 | 9686.3 ± 2382.7 | 145.5 ± 37.7 | 15887.9 ± 5409 | 399.8 ± 69.7 | 1.8 ± 1.0 |
| 100 | 304.7 ± 118.2 | 9750.2 ± 2813.6 | 137.8 ± 32.3 | 15394.2 ± 5040.3 | 352.1 ± 46.7 | 3.2 ± 1.1 |
| 1000 | 312.5 ± 119.0 | 10173.0 ± 2962.4 | 169.7 ± 40.4 | 18047 ± 4065.7 | 405.7 ± 75.9 | 1.7 ± 0.9 |
| **Chemerin (ng.mL^-1^)** | n=6 | n=6 | n=6 | n=6 | n=6 | n=5 |
| 0 | 245.6 ± 84.3 | 13900.7 ± 5176.6 | 142.7 ± 35.0 | 20983.1 ± 13165.1 | 905.9 ± 407.7 | 4.6 ± 3.1 |
| 10 | 213.7 ± 100 | 12059.9 ± 4384.0 | 89.6 ± 24.5 | 20851.7 ±12821.5 | 969.3 ± 442.9 | 6.9 ± 6.1 |
| 100 | 167.7 ± 71.2 | 11909.5 ± 3519.4 | 112.6 ± 32.0 | 22115.2 ± 13114.8 | 789.2 ± 431.3 | 0.9 ± 0.4 |
| 1000 | 192.3 ± 57.4 | 12855.3 ± 4775.3 | 121.2 ± 27.7 | 22616.3 ± 12985.2 | 941.7 ± 370.2 | 4.8 ± 3.9 |
| **Visfatin (ng.mL-1)** | n=5 | n=5 | n=5 | n=5 | n=5 | n=5 |
| 0 | 100.4 ± 29.8 | 4832.6 ± 1228.9 | 127.8 ± 44.0 | 17334.8 ± 463.7 | 486.0 ± 154.4 | 5.1± 2.5 |
| 50 | 42.1 ± 6.6 | 4483.5 ± 1637.5 | 67.7 ± 14.9 | 20910.4 ± 4081.6 | 373.0 ± 88.3 | 2.4 ± 1.5 |
| 100 | 156.8 ± 90.0 | 4546.3 ± 1106.7 | 90.8 ± 8.2 | 23690.3 ± 5744.6 | 389.1 ± 93.8 | 3.0 ± 1.4 |
| 250 | 65.9 ± 18.0 | 4454.0 ± 1297.4 | 95.1 ± 23.1 | 23358.9 ± 4607 | 341.5 ± 107.6 | 2.7 ± 1.6 |
| 500 | 121.2 ± 64.1 | 4411.5 ± 950.2 | 116.6 ± 33.5 | 17339.8 ± 5511.7 | 364.4 ± 94.9 | 1.2 ± 0.7 |
| **B.** |  |  |  |  |  |  |
|  | **TNF-α (50ng.mL^-1^)** | | | | | |
| Adipokines | IL-6 | CXCL-8 | CCL-2 | CXCL-1 | CCL20 | CCL5 |
| **Leptin (ng.mL^-1^)** | n=8 | n=9 | n=8 | n=7 | n=8 | n=8 |
| 0 | 694.7 ± 295.6 | 16905.6 ± 2886.9 | 376.2 ± 143.1 | 22903.7 ± 9783.6 | 1592.7 ± 673.6 | 14.2 ± 3.4 |
| 1 | 464.0 ± 135.1 | 15030.0 ± 2464.3 | 497.8 ± 247.9 | 26266.6 ± 10986.7 | 1732.5 ± 1017.2 | 12.8 ± 7.1 |
| 10 | 154.4 ± 129.2 | 15457.3 ± 2518.9 | 583.3 ± 267.8 | 29211.1 ± 11809.0 | 1725.5 ± 888.3 | 20.6 ± 6.5 |
| 100 | 541.2 ± 137.1 | 16152.4 ± 2365.6 | 580.4 ± 263.1 | 31152.6 11496.9 | 1971.4 ± 1145.5 | 15.6 ± 7.3 |
| 1000 | 447.5 ± 118.2 | 16363.9 ± 2588.8 | 673.4 ± 321.6 | 26671.4 ± 10223.5 | 2115.8 ± 1280.1 | 16.9 ± 5.6 |
| **Chemerin (ng.mL^-1^)** | n=6 | n=6 | n=6 | n=6 | n=6 | n=5 |
| 0 | 338.0 ± 67.7 | 17540.0 ± 4231.0 | 275.9 ± 107.1 | 31945.4 ± 17831.9 | 2344.2 ± 1126.5 | 18.6 ± 3.2 |
| 10 | 303.9 ± 64.2 | 15769.5 ± 3971.0 | 334.2 ± 160.9 | 34109.6± 17199.6 | 2746.0 ± 1439.0 | 11.8 ± 8.3 |
| 100 | 307.6 ± 68.2 | 15929.3 ± 3446.0 | 425.1 ± 256.0 | 33433.7 ± 18479.4 | 3014.9 ± 1646.6 | 5.3 ± 4.9 |
| 1000 | 295.3 ± 70.8 | 15214.2 ± 3676.5 | 397.7 ± 265.3 | 33274.0 ± 18772.3 | 2662.0 ± 1445.8 | 8.0 ± 4.2 |
| **Visfatin (ng.mL-1)** | n=5 | n=5 | n=5 | n=5 | n=5 | n=4 |
| 0 | 413.8 ± 183.0 | 13178.2 ± 2822.9 | 568.8 ± 179.9 | 31027.0 ± 11964.6 | 2283.3 ± 975.1 | 22.3 ± 3.1 |
| 50 | 452.5 ± 273.5 | 14759.1 ± 3109.9 | 829.5 ± 379.8 | 42027.2 ± 13397.1 | 2072.5 ± 872.1 | 20.4 ± 7.5 |
| 100 | 387.1 ± 183.8 | 14265.6 ± 3022.5 | 1008.0 ± 447.0 | 41711.8 ± 12578.9 | 2145.0 ± 815.1 | 24.7 ± 8.7 |
| 250 | 361.2 ± 172.5 | 14712.3 ± 2957.2 | 740.7 ± 358.5 | 44118.4 ± 14159.9 | 2187.8 ± 844.7 | 24.5 ± 8.1 |
| 500 | 370.6 ± 173.0 | 13061.9 ± 3352.9 | 1027.8 ± 544.6 | 34832.5 ± 1419.2 | 2123.5 ± 1222.2 | 20.4 ± 6.8 |

| **C.** |  |  |  |  |  |  |
| --- | --- | --- | --- | --- | --- | --- |
|  | **poly(I:C) (10µg.mL^-1^)** | | | | | |
| Adipokines | IL-6 | CXCL-8 | CCL-2 | CXCL-1 | CCL20 | CCL5 |
| **Leptin (ng.mL^-1^)** | n=5 | n=5 | n=5 | n=5 | n=5 | n=5 |
| 0 | 16848.4 ± 2333.8 | 197796.7 ± 46751 | 9213.7 ± 2229.8 | 181169.3 ± 45941.5 | 12825.9 ± 4124.7 | 8454.8 ± 1812.5 |
| 1 | 16358.1 ± 2412.3 | 175123.0 ± 54478.2 | 7042.8 ± 1424.6 | 179197.6 ± 49196.6 | 11250.0 ± 4497.3 | 8243.2 ± 1975.5 |
| 10 | 18695.0 ± 2532.4 | 185183.8 ± 47292.0 | 9565.5 ± 2133.2 | 174990.6 ± 55316.6 | 12156.7 ± 3621.2 | 8223.1 ± 1823.8 |
| 100 | 18815.2 ± 2496.6 | 187121.1 ± 41475.7 | 9249.3 ± 2073.5 | 184071.8 ± 60274.7 | 13139.5 ± 3804.2 | 9336.6 ± 1976.3 |
| 1000 | 17288.3 ± 2018.9 | 189311.0 ± 49376.5 | 8826.9 ± 2437.0 | 186209.2 ± 58922 | 13231.2 ± 3866.9 | 8146 ± 1732 |
| **Chemerin (ng.mL^-1^)** | n=5 | n=5 | n=5 | n=5 | n=5 | n=5 |
| 0 | 16848.4 ± 2333.8 | 197796.7 ± 46751.0 | 9213.7 ± 2229.8 | 181169.3 ± 45941.5 | 12825.9 ± 4124.7 | 8454.8 ± 1812.5 |
| 10 | 17298.2 ± 2668.0 | 175405.8 ± 46530.0 | 9657.9 ± 2273.2 | 160452.9 ± 46416.7 | 10426.8 ± 3559.7 | 8650.5 ± 1791.3 |
| 100 | 16389.2 ± 2245.5 | 181630.1 ± 47261.7 | 8948.4 ± 1736.9 | 159074.9 ± 39279.2 | 10676.7 ± 3804.9 | 8869.1 ± 1688.1 |
| 1000 | 16538.9 ± 2242.4 | 180001.2 ± 45652.9 | 7701.0 ± 1705.3 | 142355.0 ± 37675.9 | 10959.3 ± 3241.2 | 8244.9 ± 1971.0 |
| **Visfatin (ng.mL-1)** | n=3 | n=3 | n=3 | n=3 | n=3 | n=3 |
| 0 | 17060.4 ± 3690.5 | 112170.2 ± 31216.0 | 3983.4 ± 257 | 109245.95±38478.1 | 15533.7 ± 20474.0 | 5214.2 ± 197.2 |
| 500 | 11295.5 ± 1312.5 | 69337.6 ± 58624.8 | 2049.5 ± 1570.2 | 75693.6 ±71034.2 | 10868.6 ± 9899.6 | 3948.3 ± 3234.6 |

**Figure S1:** The level of expression of the AdipoRs was assessed in human bronchial rings crushed and homogenized in TRIzol^®^ reagent immediately after dissection using a TissueLyser LT ball mill (Qiagen Courtaboeuf, France). We have compared the expression of the AdipoRs in paired bronchial rings with an intact epithelium (Epi+) and without epithelium (Epi-) after gentle scratching. The results suggest that the epithelium accounts for about 50% of the AdipoR expression in isolated human bronchial rings.
